# Autonomic Dysregulation Attributed to Vertebral Artery Insufficiency Secondary to Subclavian Steal Syndrome: A Case Report

**DOI:** 10.7759/cureus.112481

**Published:** 2026-07-11

**Authors:** Lara Zargarian, Hoang Nguyen, Manbir Sanghera, Arman Megerdechyan, William S Hughes

**Affiliations:** 1 Medicine, Kansas City University, Joplin, USA; 2 Family Medicine, Freeman Health System, Joplin, USA

**Keywords:** autonomic dysregulation, peripheral circulation, retrograde bloodflow, subclavian steal syndrome, vertebral artery insufficiency

## Abstract

Anatomic malformation as seen in subclavian steal syndrome leads to an alteration in the body's autonomic regulatory pattern, influencing varying physical manifestations. The autonomic nervous system provides situational regulatory feedback to the body, maintaining the body’s desired point of homeostasis. We report the case of a 68-year-old male who initially came in with X-rays indicating retrolisthesis and calcifications in his carotids. A left-sided carotid bruit was also noted, as well as right-sided upper extremity weakness. Continued imaging studies indicated structural changes to his cervical regions and continued calcifications in the remainder of his arterial supply branching off of his carotid arteries. The patient's denial of surgical intervention led to treatments focused on symptomatic control until more conclusive data could be collected and interpreted.

## Introduction

Subclavian steal syndrome (SSS) is a phenomenon characterized by stenosis or occlusion of the subclavian artery proximal to the origin of the vertebral artery, resulting in retrograde hemodynamics within the ipsilateral vertebral artery [1]. Under normal physiological conditions, blood flows from the aorta into the subclavian artery and to the vertebral artery, supplying oxygenated blood to the brainstem and cerebellum [1,2]. Nevertheless, with significant stenosis or occlusion of the subclavian artery proximal to the vertebral origin, there is an obstruction of flow to the ipsilateral upper extremity [2]. To compensate, blood from the contralateral vertebral artery can pass through the Circle of Willis and reverse direction within the ipsilateral vertebral artery, thereby supplying the affected arm rather than the posterior cerebral circulation [1]. The etiology of the disease is linked to atherosclerosis, with its prevalence ranging between 0.6% and 6.4% [2]. Approximately 5.3% of those patients present with neurologic symptoms [2]. A key diagnostic point of the disease is a significant difference in blood pressure between the two arms, typically presenting as a variation of 20 mmHg or greater, along with arm claudication [3,4]. Disease severity is classified in accordance with a three-tier naming system based on blood flow and progression of arterial stenosis [4]. 

While the majority of patients who have SSS are asymptomatic, the minority present with symptoms that resemble those of claudication and autonomic dysregulation [4,5]. Arm claudication, one of the most common manifestations in symptomatic patients, is one of the major contributors to the blood pressure discrepancy and can be a marker for the severity of the disease [3]. In an attempt to compensate for the disruption in its standard blood flow, the body creates retrograde blood flow patterns in the vertebral artery [6]. Retrograde flow in the vertebral artery leads to an interruption in the correlated collateral circulation in the Circle of Willis, specifically affecting the connection between the vertebral artery and posterior communicating artery (PCA) [7]. As the vertebral artery system feeds into the vestibular system and peripheral and central auditory system, this interruption manifests neurotological symptoms seen in vertebrobasilar insufficiency (VBI) such as vertigo and more extremity-centric signs such as hypoesthesia and paresthesia [7,8]. This report describes the autonomic repercussions that may arise as a result of SSS and its associated pathologies.

This work has not been previously presented or published in abstract form at any professional conference.

## Case presentation

A 68-year-old man diagnosed with SSS in 2026 was followed by family medicine. The patient was managed at Freeman Family Medicine Associates in Joplin, Missouri, USA. His medical history includes a family history of hypertension, hypercholesterolemia, and cardiovascular disease. These factors contribute to the build-up of plaque within the arteries leading to blockages. He is also a current tobacco user, using about 3/4 ppd (pack per day) since the age of 20 in the form of cigarettes. The patient has a remote history of hepatitis A infection, skin tags, two cervical fusions in 1999 and 2002 at C5-6 and C6-7, meniscal repair in 2012, closed head injury, and hyperlipidemia. The medications he is currently taking include multivitamins (every day by mouth), fish oil (1000 mg every day by mouth), acetaminophen as needed, atorvastatin (20 mg every day by mouth), tadalafil (20 mg by mouth as needed prior to intercourse), and gabapentin (100 mg twice daily by mouth)

In December of 2025, the patient was referred to radiology to obtain X-rays of the cervical region due to his continuous neck pain. The head and neck X-ray results showed retrolisthesis at C3-4 and C4-5 with exaggeration on extension views and reduction on flexion views. Calcifications in the carotids were also noted as well as a left-sided carotid bruit. Following his imaging, the patient was seen in office in January of 2026, where he described right-sided weakness, a right distal upper extremity throbbing sensation, and radiation down the right arm into the digits. As a result of these initial physical findings, the patient was instructed to begin taking 81 mg of aspirin and obtain a carotid duplex as well as an MRI. 

**Figure 1:**
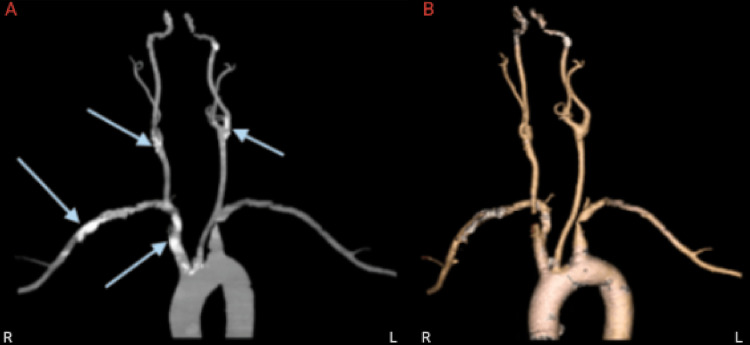
Areas of calcification found on carotid duplex study (blue arrows). (A and B) Carotid duplex study images highlighting the areas of calcification and blockage noted in the imaging report. Blue arrows indicate areas of greater calcification concentration. Areas highlighted are areas noted for significant stenosis which contribute to a decrease in blood flow and an increase in blood pressure.

Vitals taken before the carotid duplex scan showed significant variation in systolic blood pressure between the right and left arm with the right arm systolic pressure measuring 140 mmHg and the left arm systolic pressure being 112 mmHg. As seen in Figure 1, there were multiple points of stenosis and plaque accumulation throughout his vasculature. The duplex study indicated that the right proximal internal carotid artery showed borderline stenosis at approximately 50% blockage, with the left proximal internal carotid artery demonstrating moderate carotid stenosis with 50% blockage of the vessel with minimal to mild intimal thickening throughout the vasculature. Along with notable retrograde left vertebral flow, there was a right subclavian peak systolic velocity of 319 cm/s with monophasic waveforms (Tables 1-2). 

**Table 1:** Right-Sided Carotid Duplex Study CCA: common carotid artery, ICA: internal carotid artery, ECA: external carotid artery, PSV: peak systolic velocity, EDV: end-diastolic velocity

Location	PSV (cm/s)	EDV (cm/s)	Plaque	Stenosis %
Prox CCA	107	-	Intimal thickening	-
Mid CCA	100	-	Intimal thickening	-
Distal CCA	102	-	Plaque noted, Heterogeneous, Minimal, Smooth, Intimal thickening	-
Bulb	-	-	Plaque noted, Heterogeneous, Mild, Smooth, Calcified, Intimal thickening	-
Prox ICA	88	25	Plaque noted, Heterogeneous, Mild, Smooth, Calcified, Intimal thickening	<50%
Mid ICA	117	43	-	-
Distal ICA	94	25	-	-
Prox ECA	136	-	-	-
Vertebral	84	-	-	-

**Table 2:** Left-Sided Carotid Duplex Study CCA: common carotid artery, ICA: internal carotid artery, ECA: external carotid artery, PSV: peak systolic velocity, EDV: end-diastolic velocity

Location	PSV (cm/s)	EDV (cm/s)	Plaque	Stenosis %
Prox CCA	128	-	Intimal thickening	-
Mid CCA	93	-	Intimal thickening	-
Distal CCA	96	-	Plaque noted, Heterogeneous, Mild, Smooth, Calcified, Intimal thickening	-
Bulb	-	-	Plaque noted, Heterogeneous, Mild, Smooth, Calcified, Intimal thickening	-
Prox ICA	129	31	Plaque noted, Heterogeneous, Mild, Smooth, Calcified, Intimal thickening	50%
Mid ICA	107	35	-	-
Distal ICA	125	39	-	-
Prox ECA	135	-	-	-
Vertebral	74	-	-	-

**Figure 2:**
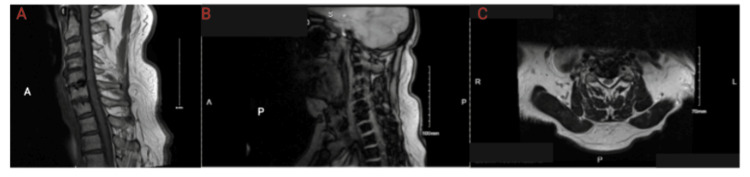
Cervical MRI Imaging Images (A-C) highlight the areas of osteophytic changes including multilevel vertebral space narrowing, central canal and foraminal narrowing, and disc osteophytic complexes.

Radiological interpretation of the C-Spine MRI indicated intervertebral disc narrowing, disc desiccation, and marginal osteophytosis (Figure 2). Imaging displayed nonspecific hyperintense T2 signals within the cervical cord at the level of C3-C4. Additionally, there were small broad-based disc osteophyte complexes, mild central canal stenosis, and bilateral mild neuroforaminal narrowing which was more pronounced in the right as opposed to the left. Imaging showed high-grade cord compression and flattening with effacement mild myelomalacia and cord flattening at C3-C4 and C4-C5. 

Following taking these images, the patient returned to the office to review his results, in which he indicated his symptoms had worsened. While some stenosis was to be expected as a result of his tobacco use and other modifiable risk factors, the degree of variation in his systolic blood pressures between his right and left arm was cause for a cardiology referral and the cervical spine findings led to a referral to spinal surgery.

During the cardiology consultation, the patient mentioned that his pain began as right hand discomfort but progressed into bilateral extremity discomfort and decreased motor activity in both upper extremities. The patient had an EKG and a stress echocardiogram done to check for any cardiologic manifestations. When compared to previous EKGs, there were no notable changes. The stress echo did indicate that the patient experienced shortness of breath, fatigue, and dizziness. When analyzing the carotid duplex study, the cardiologist noted a 30 mm decrease between right and left systolic pressure, confirming the initial diagnosis of SSS.

After the patient's cardiology consultation, he went for an evaluation with a spinal surgery specialist. A cervical exam was completed and the specialist noted positive Hoffman's sign at the right and left, centrally positive Spurling maneuver, and mild tenderness to palpation of the cervical spinal region with limited range of motion. Motor exam revealed a mildly antalgic gait and decreased bilateral upper extremity strength. Sensory exam identified decreased sensation to pin along the entire right and left upper extremities as well as decreased sensation to light touch along the entire right and left upper extremities. Examination of his reflexes showed increased but equal deep tendon reflexes in the upper and lower extremities, right and left biceps tendon reflex +3, right and left brachioradialis deep tendon reflex of +3, and right knee deep tendon reflex +3 with a left knee deep tendon reflex of +2. A sustained clonus was also noted in both the right and left extremities. Peripheral vasculature examination showed brisk capillary refill on his evaluation as well. 

Based on the results, surgery was ultimately recommended and discussed by the orthopedic spinal surgery team with the goal of relieving some of the symptoms caused by the stenosis in his vertebrae. The patient was advised to wear a brace before and after surgery to help with overall stabilization of his back until he is cleared by his surgical team. Additionally, unless told otherwise, the patient was advised to continue his current medication regimen. 

## Discussion

This case demonstrates the correlation between the interconnected systems within the body and the manifestations that arise from deferment of a homeostatic state. Although considered an uncommon manifestation of SSS, autonomic dysregulation is thought to result from vertebrobasilar hypoperfusion secondary to proximal subclavian artery stenosis [9]. This hypoperfusion is hypothesized to contribute to the neurologic and autonomic manifestations that are associated with VBI [9].

**Figure 3:**
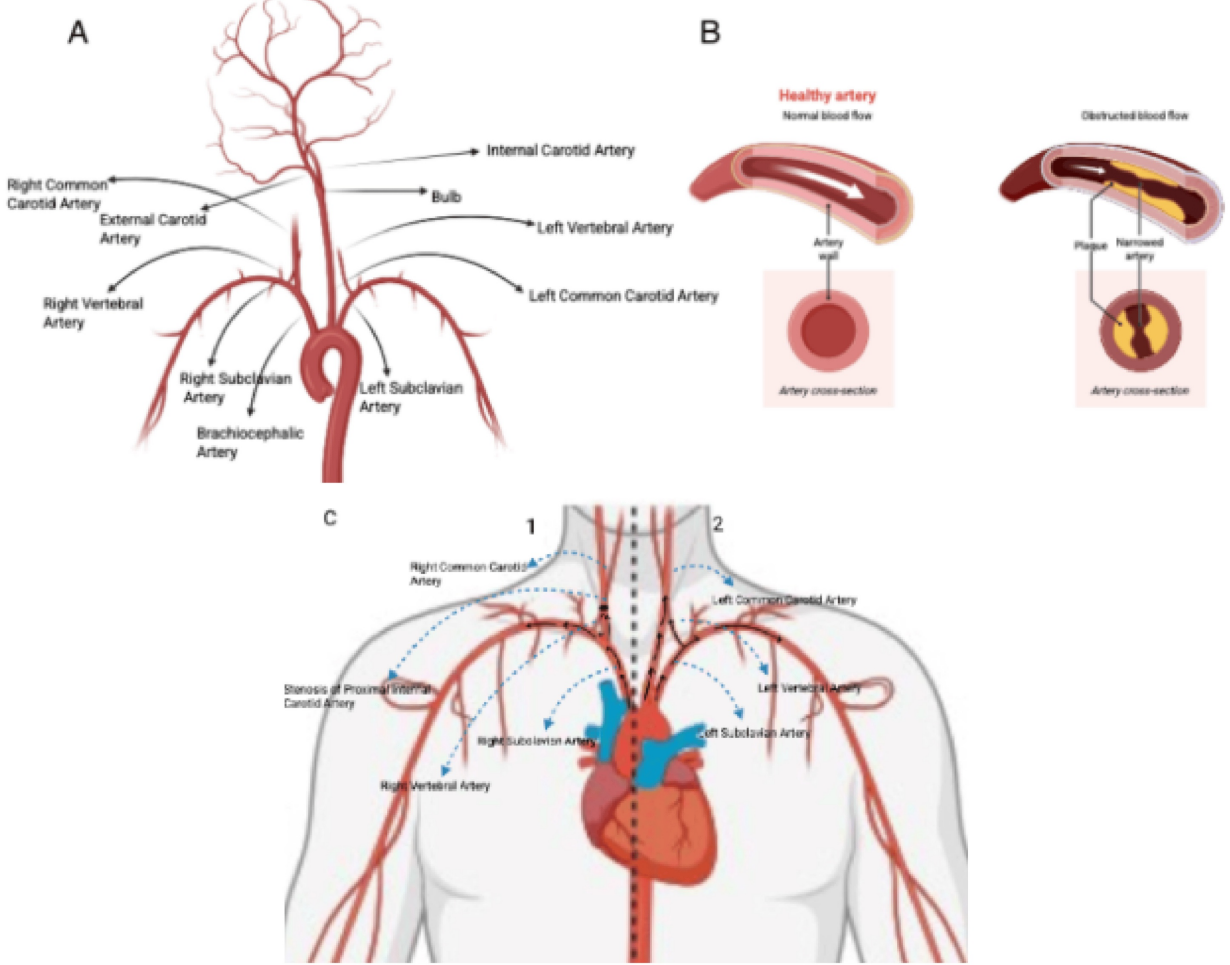
Standard Vascular Structure With Pathologic Changes (A) Demonstrates the standard anatomical composition of the vascular structure leading directly off of the heart through the upper chest and up the neck. (B) Shows the pathological changes that occur in patients experiencing coronary artery disease. The image shows the pathologic change that occurs within the artery as plaque and calcifications build and grow over time. (C) 1) Indicates a blockage of the proximal internal carotid artery and demonstrates how the blood flow results in retrograde flow as a result of the blockage. 2) Demonstrates the normal vasculature before a stenosis develops and leads to the retrograde blood flow seen in the disease process. Arrows and base pictures were generated using BioRender. Image editing and labeling credit: Lara Zargarian, Author

The acute angle of the left subclavian artery, along with associated environmental factors, result in increased turbulent flow and expedite the development of atherosclerosis at the left subclavian branch point [2,4,9]. As seen in Figure 3, the vertebral artery is a branch point off of the subclavian artery. As the vertebral artery progresses further up into the brain, it comes together to form the basilar artery and the posterior portion of cerebral circulation [9]. This circulatory complex supplies critical structures of the brain such as the brainstem, cerebellum, and occipital lobes which control unconscious functions of daily living [9]. Due to the anatomical relationship, a blockage of vasculature within this region can lead to the phenomenon of retrograde blood flow (Figure 3). As a result, internal brainstem structures such as the medulla, which contains the nucleus tractus solitarius (NTS), will have difficulty detecting changes in blood pressure and induce a change in heart rate in response [4,10]. Should retrograde flow intensify, collateral flow to other parts of the body decreases and can lead to the development of peripheral symptoms varying in physiological origin [6]. 

In addition to the potential autonomic effects of vertebrobasilar hypoperfusion, other sensory manifestations regulated by the autonomic nervous system (ANS) may develop such as dysesthesia. Although dysesthesia is not a standard manifestation of SSS, it can present itself in symptomatic patients [2]. Presentation includes arm pain, fatigue, numbness, or paresthesias which are considered secondary to upper extremity ischemia and arterial insufficiency [2,11,12]. The patient's presentation of right hand pain, decreased motor activity, and tingling sensation along the upper extremities can be attributed to the blockage of blood flow to the region as a result of the stenosis in his vessels. These symptoms may be attributed to some of the key signs of acute limb ischemia known as the Six Ps: pain, pallor, paraesthesia, paralysis, pulselessness, and poikilothermia [13]. Risk factors for acute limb ischemia are hypertension, hypercholesterolemia, hyperlipidemia, and tobacco use, all of which the patient has a family history or past medical history of predisposing him for peripheral vascular changes originating from the lack of proper circulation in a more proximal region. 

The ANS’s influence on the cardiovascular system can lead to disruptions of proper regulation of vascular tone, blood pressure, and heart rate [14]. When vascular tone is affected, patients may experience edema in both upper and lower extremities due to venous pooling, decreased venous return, and increased capillary hydrostatic pressure with the possibility of fluid retention [15,16]. Signs such as dizziness and exercise intolerance are possible indicators that there may be a form of autonomic dysregulation taking place known as dysautonomia [17]. When put into a situation that required proper blood flow such as the cardiac stress echo, the patient experienced dizziness because blood was not able to flow through the proper channels and perfuse all the necessary structures, intensifying the already present diversion of blood. 

For symptomatic patients, surgical intervention such as carotid-subclavian bypass, axillo-axillary bypass, or percutaneous transluminal angioplasty of the subclavian artery with stent placement is the most direct way to see a difference in presumed autonomic pathologic manifestations [7]. The goal of surgical intervention is to restore anterograde vertebral flow, which would improve symptoms associated with VBI [7,9]. Long-term care includes monitoring blood pressure and cholesterol levels as well as routine imaging to view the progression of plaque buildup and exacerbation of stenosis [2].

Limitations

This report is limited by a single-patient design, limiting its overall generalizability. There was no specific autonomic testing done on the patient. Due to the lack of testing, the relationship between the patient’s clinical presentation and vertebrobasilar hypoperfusion is hypothesized instead of being proven with a causative relationship. Until the patient is able to get surgical relief, his care plan is restricted to secondary prevention methods including medications such as gabapentin and aspirin to mediate the symptoms the patient is experiencing. 

The patient has a history of high-grade cord compression with mild myelomalacia. High-grade cord compression is caused by compression of the spinal cord which may result in myelomalacia, a disruption of nerve function [18]. This dysfunction can result in the right-sided weakness and radiation of throbbing sensation the patient described which created a limitation in knowing whether some of the symptoms the patient presented with were a direct result of his cervical issues compressing on nerve roots or from the theorized autonomic dysregulation that may have resulted from the vascular insufficiency. 

However, the patient did present with cervical symptoms years prior to symptom presentation of SSS, making the causal effect less direct and more circumstantial. While the cervical compression may have contributed to weakness and throbbing, it does not address the complication of blood pressure and blood flow that the patient presented with. Blood vessels are lined by endothelial cells and they play a crucial role in managing the vasculature. Additionally, the endothelium has the ability to affect the ANS through vasoactive factors such as nitric oxide (NO), the release of which can affect the parasympathetic nervous system, preventing its activation and thereby affecting blood flow to organs, impairing their function and exacerbating physical symptomology [10,19].

## Conclusions

Autonomic dysregulation secondary to SSS can mimic various conditions leading to some challenges in the treatment course for symptomatic patients. While cervical issues may contribute to autonomic dysfunction due to nerve damage, the stenosis in the patient's vessels that resulted in retrograde blood flow is thought to have a greater impact on autonomic dysregulation throughout the body as a whole. This case provided insight into the relationship between various body systems and showed how the slightest degree of change can completely rearrange the body's natural homeostatic state.
